# Total neoadjuvant therapy for the treatment of locally advanced rectal cancer: a systematic minireview

**DOI:** 10.1186/s13062-022-00329-7

**Published:** 2022-06-13

**Authors:** Andrea M. Guida, Bruno Sensi, Vincenzo Formica, Rolando M. D’Angelillo, Mario Roselli, Giovanna Del Vecchio Blanco, Piero Rossi, Gabriella T. Capolupo, Marco Caricato, Giuseppe S. Sica

**Affiliations:** 1 Department of Surgery, Minimally Invasive Unit, University of Rome Tor Vergata, 00133 Rome, Italy; 2 Department of Systems Medicine, Medical Oncology Unit, Policlinico Tor Vergata, Rome, Italy; 3 Department of Biomedicine and Prevention, Radiation Oncology, University of Rome Tor Vergata, 00133 Rome, Italy; 4 Department of Systems Medicine, Gastroenterology Unit, University of Rome Tor Vergata, 00133 Rome, Italy; 5 Department of Colorectal Surgery, Colorectal Surgery Unit, Campus Bio-Medico University, 00128 Rome, Italy; 6 Department of Surgery, Policlinico Tor Vergata, University of Rome, Tor Vergata, Viale Oxford 81, 00133 Rome, Italy

**Keywords:** Total Neoadjuvant Therapy (TNT), Neoadjuvant chemoradiotherapy, Rectal cancer, Locally Advanced Rectal Cancer (LARC), Tumor Regression Grade (TRG), Pathological Complete Response (pCR)

## Abstract

Colorectal carcinoma is the second leading cause of cancer-related deaths, and indeed, rectal cancer accounting for approximately one third of newly diagnosed patients. Gold standard in the treatment of rectal cancer is a multimodality approach, aiming at a good control of the local disease. Distant recurrences are the major cause of mortality. Currently, Locally Advanced Rectal Cancer (LARC) patients undergo a combined treatment of chemotherapy and radiotherapy, followed by surgery. Eventually, more chemotherapy, namely adjuvant chemotherapy (aCT), may be necessary. Total Neoadjuvant Therapy (TNT) is an emerging approach aimed to reduce distant metastases and improve local control. Several ongoing studies are analyzing whether this new approach could improve oncological outcomes. Published results were encouraging, but the heterogeneity of protocols in use, makes the comparison and interpretation of data rather complex. One of the major concerns regarding TNT administration is related to its effect on larger and more advanced cancers that might not undergo similar down-staging as smaller, early-stage tumors. This minireview, based on a systematic literature search of randomized clinical trials and meta-analysis, summarizes current knowledge on TNT. The aim was to confirm or refute whether or not current practice of TNT is based on relevant evidence, to establish the quality of that evidence, and to address any uncertainty or variation in practice that may be occurring. A tentative grouping of general study characteristics, clinical features and treatments characteristics has been undertaken to evaluate if the reported studies are sufficiently homogeneous in terms of subjects involved, interventions, and outcomes to provide a meaningful idea of which patients are more likely to gain from this treatment.

## Introduction

**Core tip:** Given the enormous amount of scientific information published every year, systematic reviews and meta-analyses have become indispensable methods for the evaluation of medical treatments and the delivery of the best evidence-based practice. Total Neoadjuvant Therapy (TNT) is an emerging approach for the treatment of Locally Advanced Rectal Cancer (LARC) aimed at improving oncological results. One of the major concerns regarding its administration is related to its effect on larger and more advanced cancers that might not undergo similar down-staging effect as smaller, early-stage tumors. For this reason, using the available evidence, we propose an interesting minireview which is also novel, ethical and relevant on this particularly complex intervention with the scope of summarizing the body of research to evaluate sources of heterogeneity.

Colorectal cancer is currently the third most prevalent cancer worldwide, with a particularly high incidence in western countries, possibly due to the concurrent increase of obesity and metabolic syndrome [1–6]. In Europe, the gold standard for the treatment of locally advanced rectal cancer (LARC) consists in a multidisciplinary approach based on the administration of either preoperative long-course chemoradiotherapy (CRT) or short-course radiotherapy (SCRT), followed by surgery and adjuvant chemotherapy [7–10].

Improved therapeutic strategies and multimodality approach have led to a better local control of the disease, thus reducing the 5 years local recurrence rate from 27 to 3.7% [11]. Preoperative radiotherapy contributed to a better control of local disease [12], but choice of the best protocol of administration and timing of surgery is still a matter of debate [7, 13–16]. Surgery has also played a major role since the introduction of total mesorectal excision [8] and, laparoscopic and robotic procedures can now be performed achieving the same oncological results in referral centers [17].

Despite several efforts and the use of a multimodality approach, distant recurrences are still significant and represent the leading cause of mortality for rectal cancer patients [18–20]. In this scenario, chemotherapy (CT) plays an important role since it could allow a better control of systemic disease, improving overall survival (OS), disease free survival (DFS) and distant recurrence rate. To date, most national guidelines include both neoadjuvant and adjuvant CT for LARC [7, 8, 21], despite lack of evidence on the true benefits of adjuvant CT (aCT) in patients who have already received neoadjuvant chemotherapy (nCT) [22–26]. However, three quarter of the patients will eventually receive aCT after surgery but less than half will complete the planned treatment [22].

Several randomized clinical trials (RCTs) aimed at optimizing LARC treatment are focusing on the intensification of neoadjuvant treatment with standard dose polychemotherapy administration before surgery, which is known as total neoadjuvant treatment (TNT). Several reasons support TNT administration:

- Potential early treatment of occult micro-metastases to improve systemic disease control
- Increase patient tolerance and compliance to CT because administered preoperatively
- Ease surgery, reducing tumor bulk and nodes
- Increase sphincter sparing procedures rate and increase organ preservation rate for patients with complete clinical response (cCR).

However, despite encouraging results, not all patients respond equally to TNT and results are variable. Existence of a spectrum of local response to TNT is well known, ranging from complete pathological response (pCR) and near-complete pathological response (npCR) to non-response. One of the major concerns regarding the administration of TNT is related to its effect on larger and more advanced rectal cancers that seem to have a worse response than smaller, early-stage tumors.

Furthermore, TNT could impact on patient performance status, thus reducing the number of patients able to tolerate surgery. In addition, non-responders can both experience micro metastases growth and local tumor progression, jeopardizing surgical treatment for those patients with previously resectable tumors. One last consideration is on potentially unnecessary overtreatments if we consider those patients that have already done well after the initial neoadjuvant treatment [27–29].

It seems therefore, important to identify factors predictive of a good response to TNT. Accurate analysis of clinical studies and treatments characteristics is necessary to evaluate interventions and outcome in order to offer a meaningful idea of which patients are more likely to gain from this treatment.

## Methods

### Literature search

A systematic literature search was carried out on Pubmed for articles published up to December 31, 2021. The following Medical Subject Headings (MeSH) terms were used: “total neoadjuvant therapy” OR “total neoadjuvant treatment” OR “neoadjuvant” AND “rectal cancer” OR “locally advanced rectal cancer”. A further search was performed in clinicaltrial.gov using the terms “total neoadjuvant therapy” and “rectal cancer”. References of the included studies were manually assessed in order to detect any missing studies.

### Inclusion and exclusion criteria

Only RCTs, systematic reviews and meta-analysis in the English language were included in the literature search. In case of duplications only the most recent or most detailed study was included. Furthermore, all selected articles had to meet all the following inclusion criteria: (1) LARC, defined as Stage II/III; (2); RCTs comparing standard CRT in the control arm versus TNT in the experimental arm.

Exclusion criteria were the following: (1) use of any additional biological drugs both in the experimental arm or in the control arm; (2) RCTs including organ preservation alone after neoadjuvant treatment.

### Data extraction

Extracted variables were the following: general study characteristics (e.g., author, name of the study, country of recruitment, year of last publication, study design, number of patients, treatment arms and primary end point), treatment protocols (RT regimens, CT agents, timing of CT administration, timing of surgery, aCT), local disease control outcomes (pCR, nodal down staging, resection, lymphovascular and perinervous invasion, local recurrence rate), distant disease control outcomes (DFS, OS, distant recurrence rate), toxicity and complications (chemo-related adverse effect, surgical complications, compliance), and predictors of disease control.

## Results of literature search

**Fig. 1:**
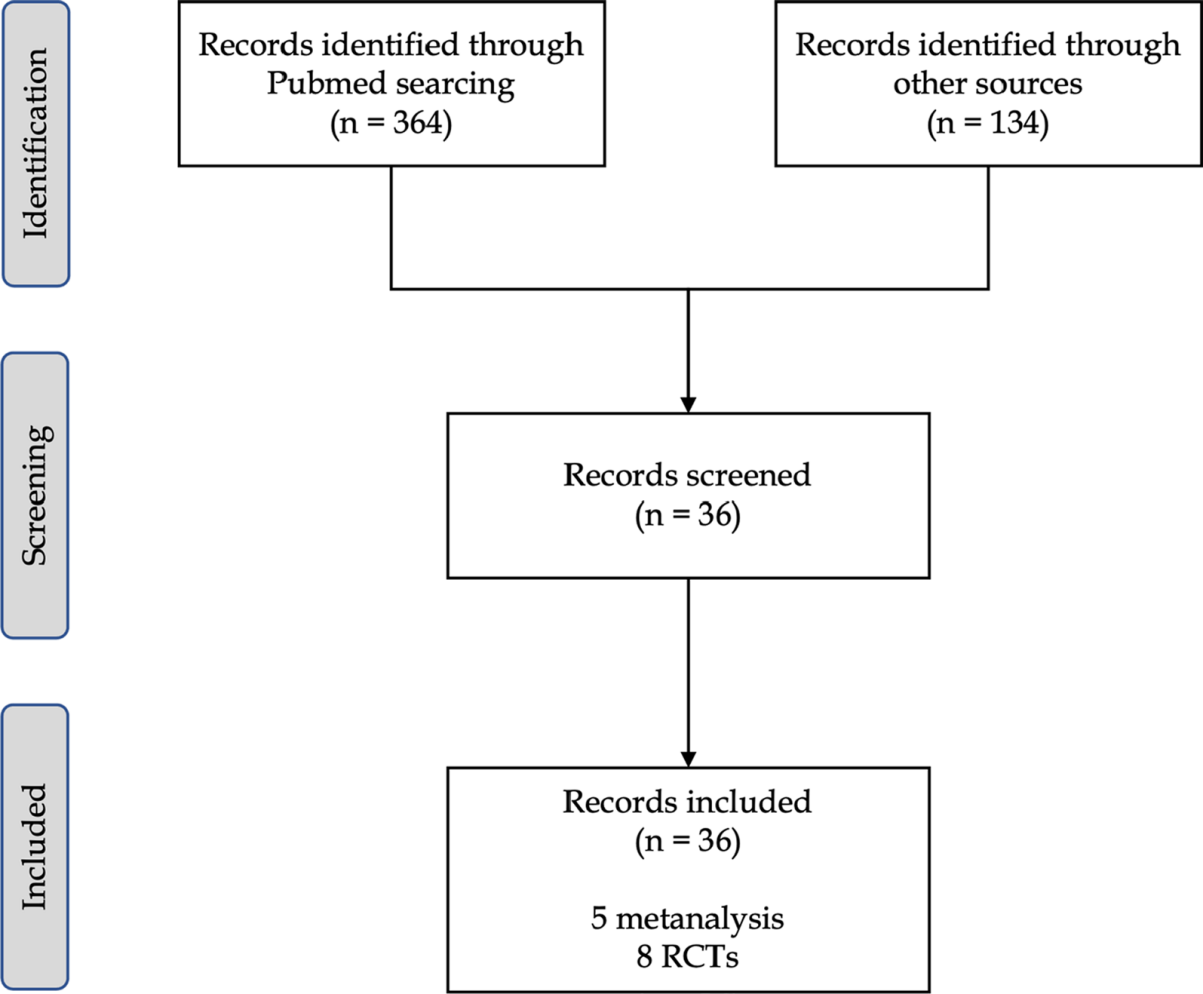
Flow diagram of included studies

Three hundred and sixty-four articles from PubMed and one hundred and thirty-four from clinicaltrial.gov were analyzed for language and article relevance depending on both title and abstract. The publication year ranged from 2011 to 2021. Five meta-analysis and eight RCTs met the inclusion’s criteria. The full detail of the inclusions process and PRISMA flow chart can be found in Fig. 1. All RCTs were included for descriptive analysis. The number of recruited patients in each study ranged from 49 to 912; totally, data regarding 2705 subjects was collected.

Collected data show wild heterogeneity in terms of RT and CT regimens, CT agents, timing of CT administration and timing of surgery for both experimental TNT and standard therapy.

## Treatment protocols for larc

Treatment protocols show high heterogeneity. In particular, protocols differ in many respects including type of radiotherapy (SCRT vs long RT plus CT), use of chemotherapeutic drugs, timing of CT administration (induction vs consolidation), timing of surgery and use of adjuvant therapy. Furthermore, differences can be found within the same study, due to the different chemo-radio therapeutic regimens employed for the two arms.

General features of the included studies are showed in Tables 1 and 2.

**Table 1:** General features of included RCTs

Author	Study	Country	Year	Study design	N	Treatment arms	Primary end point
Marèchal R	EudraCT	Belgium	2011	Randomized phase II	57	**(Exp arm):** FOLFOX × 2 → CRT (5 FU) → Sx **(Control):** CRT (5 FU) → Sx	pCR
Fernandez-Martos C	GCR-3	Spain	2015	Randomized phase II	108	**(Exp arm):** CAPOX × 4 → CRT (CAPOX) → Sx **(Control):** CRT (CAPOX) → Sx → CAPOX	pCR
Bujko K	POLISH II	Poland	2016	Randomized phase III	515	**(Exp arm):** SCRT → FOLFOX × 2 → Sx **(Control):** CRT (FOLFOX × 3) → Sx	R0 resection rate
Moore J	WAIT	Australia	2017	Randomized phase II	49	**(Exp arm):** CRT (5FU) → 5 FU × 3 → Sx **(Control):** CRT (5FU) → Sx	pCR
Kim SY	KCSG CO 14-03	Korea	2018	Randomized phase II	108	**(Exp arm):** CRT (capecitabine) → CAPOX × 2 → Sx **(Control):** CRT (capecitabine) → Sx	Downstaging rate
Deng Y	FOWARC	China	2019	Randomized phase III	495	**(Exp arm):** mFOLFOX6 + CRT (5FU) → Sx → 5FU × 7 **(Exp arm):** mFOLFOX6 × 4–6 → Sx → mFOLFOX6 × 6–8 **(Control):** CRT (5FU) → Sx → 5FU × 7	3-yr DFS
Bahadoer RR	RAPIDO	Netherlands	2020	Randomized phase III	912	**(Exp arm):** SCRT → CAPOX × 6/FOLFOX × 9 → Sx **(Control):** CRT (capecitabine) → Sx → CAPOX × 6/FOLFOX4 × 12	DrTF
Conroy T	PRODIGE-23	France	2021	Randomized phase III	461	**(Exp arm):** FOLFIRINOX × 6 → CRT (capecitabine) → Sx → FOLFOX × 6 **(Control):** CRT (capecitabine) → Sx → FOLFOX × 12	3-yr DFS

**Table 2:** General features of included metanalysis

Author	Year	Inlcuded studies (N)	Included RCT (N)	Patients (N)
Peterlli F	2019	28	5	3579
Riesco-Martinez MC	2020	8	8	2301
Kasi A	2020	7	4	2416
Kong JC	2021	15	7	2437
Liu S	2021	8	8	2196

**Table 3:** Baseline features of eligible patients. c T stage: clinical stage; c N stage: clinical N stage; EMVI; extramural vascular invasion; MRF: mesorectal fascia involment

Study	ARMS (N patients)	Age mean (range)	cT Stage N (%)			cN Stage N (%)			Enlarged lateral nodes N (%)	EMVI + N (%)	MRF + N (%)	Predicted CRM + N (%)		
			cT2	cT3	cT4	cN0	cN1	cN2				≤ 1 mm	> 1 mm	NR
EudraCT	EXP. ARM (28)	62 (22–80)	1 (4%)	25 (89%)	2 (7%)	–	26 (93%)		–	–	–	7 (25%)*	18 (64%)**	3 (11%)
	CONTROL (29)	62 (44–79)	3 (10%)	23 (79%)	3 (10%)		25 (86%)					9 (31%)*	15 (52%)**	5 (17%)
GCR-3	EXP. ARM (56)	60 (38–76)	–	18 (32%)	7 (13%)	–	31 (55%)		–	–	–	–	–	–
	CONTROL (52)	62 (42–75)		12 (23%)	3 (6%)		31 (59%)				5 (10%)			
POLISH II	EXP. ARM (261)	60	–	88 (34%)	165 (63%)	–	–	–	–	–	–	–	–	–
	CONTROL (254)	60		83 (33%)	163 (64%)									
WAIT	EXP. ARM (25)	60	0	24 (96%)	1 (4%)	0	6 (24%)	19 (76%)	–	–	7 (28%)	–	–	–
	CONTROL (24)	61	1 (4%)	18 (75%)	5 (21%)	2 (8%)	7 (30%)	15 (62%)			8 (33%)			
KCSG CO 14-03	EXP. ARM (53)	56	–	44 (81%)	9 (19%)	3 (6%)	49 (92%)		–	–	14 (26%)	–	–	–
	CONTROL (55)	55		45 (82%)	10 (18%)	4 (7%)	51 (93%)				16 (29%)			
FOWARC	ARM TNT + RT (165)	52	3 (2%)	106 (64%)	56 (34%)	–	88 (53%)	47 (29%)	–	–	38/107 (36%)	–	–	–
	ARM TNT (165)	54	1 (1%)	114 (70%)	50 (30%)		76 (41%)	43 (26%)			22/105 (31%)			
	CONTROL (165)	54	8 (5%)	100 (61%)	57 (35%)		84 (51%)	44 (26%)			32/101 (32%)			
RAPIDO	EXP. ARM (462)	62 (31–83)	14 (3%)	301 (65%)	147 (32%)	42 (9%)	118 (26%)	302 (65%)	66 (14%)	148 (32%)	285 (62%)	–	–	–
	CONTROL (450)	62 (23–84)	14 (3%)	299 (66%)	137 (30%)	35 (8%)	120 (27%)	295 (66%)	69 (15%)	125 (28%)	271 (60%)			
PRODIGE-23	EXP. ARM (231)	61 (34–77)	3 (1%)	182 (81%)	40 (18%)	24 (10%)	148 (64%)	59 (26%)	23 (10%)	–	–	48 (21%)	137 (59%)	46 (20%)
	CONTROL (230)	62 (26–75)	2 (1%)	188 (84%)	35 (16%)	22 (10%)	155 (67%)	53 (23%)	24 (10%)			54 (23%)	141 (61%)	35 (15%)

*CRM ≤ 5 mm. **CRM < 5 mm

**Table 4:** Pathologoical outcomes, first part

Study	ARMS (N patients)	ypT stage N (%)						ypN stage N (%)				pCR N (%)	Local recurrence N (%)	Distant recurrence N (%)
		ypT0	ypTis	ypT1	ypT2	ypT3	ypT4	ypN0	ypN1	ypN2	ypNx			
EudraCT	EXP. ARM (28)	7 (25%)	1 (4%)	1 (4%)	4 (14%)	13 (46%)	1 (4%)	13 (46%)	9 (32%)	5 (18%)	–	7 (25%)	–	–
	CONTROL (29)	8 (28%)	1 (3%)	1 (3%)	5 (17%)	12 (41%)	1 (3%)	16 (55%)	9 (31%)	3 (10%)		8 (28%)		
GCR-3	EXP. ARM (56)	*	*	*	*	*	*	*	*	*	*	8 (14%)	3 (5%)	13 (23%)
	CONTROL (52)											7 (13%)	1 (2%)	11 (21%)
POLISH II	EXP. ARM (261)	24 (12%)	–	5 (3%)	53 (26%)	92 (46%)	28 (14%)	136 (68%)	37 (19%)	27 (14%)	–	24 (12%)	–	62 (25%)
	CONTROL (254)	37 (17%)		3 (1%)	47 (22%)	110 (51%)	19 (9%)	150 (69%)	43 (20%)	26 (12%)		37 (17%)		75 (29%)
WAIT	EXP. ARM (25)	5 (20%)	–	1 (4%)	5 (20%)	13 (52%)	1 (4%)	16 (73%)	5 (20%)	4 (16%)	–	–	–	–
	CONTROL (24)	6 (25%)		2 (8%)	4 (17%)	11 (46%)	1 (4%)	19 (79%)	2 (8%)	3 (13%)				
KCSG CO 14-03	EXP. ARM (53)	6 (14%)	0	1 (2%)	12 (27%)	24 (55%)	1 (2%)	28 (64%)	15 (34%)	1 (2%)	–	6 (14%)	–	–
	CONTROL (55)	3 (6%)	1 (2%)	1 (2%)	8 (15%)	39 (75%)	0	27 (52%)	20 (28%)	5 (10%)		3 (6%)		
FOWARC	ARM TNT + RT (165)	84 (87%)			65 (44%)			–				41 (27%)	–	–
	ARM TNT (165)	54 (35%)			98 (65%)							10 (7%)		
	CONTROL (165)	53 (37%)			90 (63%)							20 (14%)		
RAPIDO	EXP. ARM (462)	129 (30%)	2 (< 1%)	17 (4%)	82 (19%)	157 (37%)	36 (9%)	317 (75%)	75 (18%)	31 (7%)	–	120 (28%)	29 (22%)	86 (67%)
	CONTROL (450)	69 (17%)	1 (< 1%)	17 (4%)	96 (24%)	190 (48%)	25 (6%)	273 (69%)	78 (20%)	47 (12%)		57 (14%)	17 (13%)	123 (81%)
PRODIGE-23	EXP. ARM (231)	60 (28%)	3 (1%)	11 (5%)	57 (27%)	77 (36%)	4 (2%)	175 (83%)	30 (14%)	7 (3%)	0	59 (28%)	–	–
	CONTROL (230)	27 (13%)	2 (1%)	17 (8%)	62 (29%)	103 (48%)	4 (2%)	145 (67%)	49 (23%)	20 (9%)	1 (1%)	26 (12%)		

Pathologoical outcomes, second part											
Study	Arms (N patients)	CRM		Resection limits N (%)			Lymphovascular invasion N (%)	Perinervous invasion N (%)	TRG N (%)		
		> 1 mm	≤ 1 mm	R0	R1	R2			1	2	3
EudraCT	EXP. ARM (28)	27 (96%)	1 (4%)	–	–	–	–	–	–	–	–
	CONTROL (29)	25 (86%)	4 (14%)								
GCR-3	EXP. ARM (56)	–	–	48 (86%)	2 (4%)	2 (4%)	–	–	–		
	CONTROL (52)			45 (87%)	1 (2%)	–					
POLISH II	EXP. ARM (261)	–	–	178 (71%)	20 (8%)	5 (2%)	–	–	–		
	CONTROL (254)			202 (77%)	17 (7%)	1 (1%)					
WAIT	EXP. ARM (25)	–	–	23 (92%)	2 (8%)	–	–	–	–		
	CONTROL (24)			22 (92%)	2 (8%)						
KCSG CO 14-03	EXP. ARM (53)	37 (84%)	7 (16%)	–			–	–	7 (16%)	24 (55%)	7 (16%)
	CONTROL (55)	47 (90%)	4 (7%)						13 (25%)	28 (54%)	7 (14%)
FOWARC	ARM TNT + RT (165)	–	–	134 (90%)	10 (7%)	5 (3%)	–	–	102 (69%)	46 (31%)	
	ARM TNT (165)			136 (89%)	8 (5%)	8 (5%)			50 (33%)	102 (67%)	
	CONTROL (165)			128 (91%)	8 (6%)	5 (3%)			70 (49%)	71 (50%)	
RAPIDO	EXP. ARM (462)	385 (91%)	38 (9%)	382 (90%)	38 (9%)	3 (1%)	–	–	–		
	CONTROL (450)	363 (91%)	35 (9%)	360 (90%)	37 (9%)	1 (< 1%)					
PRODIGE-23	EXP. ARM (231)	149 (95%)	8 (5%)	201 (95%)	20 (5%)	0	17 (8%)	15 (7%)	88 (48%)	71 (38%)	26 (14%)
	CONTROL (230)	173 (94%)	11 (6%)	202 (94%)	10 (5%)	2 (1%)	20 (9%)	24 (11%)	57 (32%)	99 (55%)	23 (13%)

yPT stage Pathologoical T stage after neuadjuvant theraphy; yPN stage Pathologoical N stage after neuadjuvant theraphy; PCR: pathological complete response. ypTNM stage; I 12 (21%) vs 21 (40%) II 18 (32%) vs 9 (17%) III 13 (23%) vs 9 (17%) IV 1 (2%) vs 0

CRM: circumferential resection margins; TRG: tumor regression grade

**Table 5:** Other outcomes

Study	ARMS (N patients)	DFS	OS	Compliance**		AEs*	Post-operative complications
				LCRT	TNT		
EudraCT	EXP. ARM (28)	–	–	23 (86%)	27 (95%)	10 (36%)	7 (25%)
	CONTROL (29)			28 (97%)	–	2 (7%)	9 (31%)
GCR-3	EXP. ARM (56)	62%^1^	75%^1^	42 (78%)	51 (94%)	12 (23%)	27 (51%)
	CONTROL (52)	64%^1^	78%^1^	46 (94%)	–	15 (29%)	21 (45%)
POLISH II	EXP. ARM (261)	53%^2^	73%^2^	–		23%	29%
	CONTROL (254)	52%^2^	65%^2^			21%	25%
WAIT	EXP. ARM (25)	–	–	–		–	–
	CONTROL (24)						
KCSG CO 14-03	EXP. ARM (53)	–	–	–		5 (9%)	4 (9%)
	CONTROL (55)					2 (4%)	1 (2%)
FOWARC	ARM TNT + RT (165)	78%^2^	90%^2^	–		–	–
	ARM TNT (165)	74%^2^	91%^2^				
	CONTROL (165)	73%^2^	91%^2^				
RAPIDO	EXP. ARM (462)	₮	89%^2^	–	84%	–	50%
	CONTROL (450)		89%^2^	93%	–		47%
PRODIGE-23	EXP. ARM (231)	76%^2^	91%^2^	219 (95%)	> 90%	73 (45%)	–
	CONTROL (230)	69%^2^	88%^2^	227 (99%)	–	117 (74%)	

DFS: Disease Free Survival; OS: Overall Survival; AEs: Adverse Effects

*Grade III/IV AEs; Compliance to the treatment is defined as administration of at least the 75% of the described dose

DrTF 30% versus 24% in the experimental and standard arms, respectively

^1^Results at five-years

^2^Results at three-years

### Radiotherapy regimens

Two different RT regimens (SCRT and CRT) are considered the standard of care for the treatment of LARC [7].

Most of the analyzed studies provided CRT both in the standard and in the experimental arm. Only two randomized phase III studies provided SCRT in the experimental arm, before the administration of consolidation CT [13, 30]. Moreover, the Chinese study by Deng et al., that used CRT for the two arms of the study, was the only one including a further experimental arm without neoadjuvant RT administration [31].

Outcomes of SCRT vs CRT are controversial. According to Kong et al. studies using SCRT reported an 86% increase in local recurrence rates compared to CRT (9.3% vs 5.3%) [27]. In contrast, Liu et al. found SCRT to lead to higher pCR rates compared to CRT [32].

CRT administration is strongly recommended if CRM and R0 resection are predicted at risk independently from T and N stage [7].

### CT agents

Continuous intravenous infusions of 5-fluorouracil (5FU) or oral capecitabine during CRT are strongly recommended [7]; on the contrary, statements regarding the administration of other CT agents are lacking or are based on low-grade recommendations (Tables 3, 4, 5).

CRT was based on single agent administration (5FU or capecitabine) in 6 out of 8 studies [30, 31, 33–36]. In all these studies CRT agents were the same for both the experimental and standard arm, except for the RAPIDO trial, which compared capecitabine-based CRT in the control arm to SCRT in the experimental arm [30]. Two RCTs used multiagent oxaliplatin-based CRT: the GCR-3 study administered capecitabine and oxaliplatin (CAPOX/XELOX) both in the experimental and standard arm [37], while the POLISH II compared 5FU/folinic acid and oxaliplatin (FOLFOX) in the control arm to SCRT in the experimental group [13].

In almost all the analyzed studies TNT consisted in the administration of multi-agent oxaliplatin-based chemotherapeutic agents. Only Moore et al. chose 5FU administration for both CRT and TNT treatment [33]. The others analyzed both CAPOX/XELOX or FOLFOX administration. The administration of 5FU/folinic acid, irinotecan and oxaliplatin (FOLFIRINOX) was used only in the PRODIGE trial [36].

The number of administered cycles and thus length of TNT widely differed too, ranging from 6 weeks for the administration of 3 cycles of 5FU in the WAIT trial [33], to 18 weeks for the administration of 6 cycles of CAPOX or 9 cycles of FOLFOX in the RAPIDO trial [30].

A recent meta-analysis of RCTs stratified pCR results depending on TNT length in terms of weeks and found that patients receiving less than 12 weeks of therapy showed no significant differences in terms of pCR rates compared to standard treatment [32].

The vast inter-study variation in CT regimens contributes to difficult interpretation of chemotherapeutic agents’ effect on primary and secondary outcomes.

### Timing of TNT administration

One of the most discussed clinical points is whether to administer TNT before or after neoadjuvant RT. In fact, induction-type TNT, which is given before neoadjuvant RT, allows an early systemic disease control, slowing occult micro metastasis growth, thus potentially reducing distant failure rates, but could determine local growth of previously resectable cancers. On the contrary, consolidation-type TNT, which is administered during the free interval between the end of neoadjuvant RT and surgery, could increase the chemoradiation-to-surgery interval but could also improve pCR after radiation and therefore increase sphincter-preserving rate.

Of the 8 RCT, four reported on induction and four on consolidation TNT.

The recent meta-analysis by Kong et al. comparing TNT to the standard of care for the treatment of LARC stratified results of both short and long-term outcomes depending on TNT type and found that induction regimens only slightly improve pCR (28%) and increase negative CRM rates but have no impact on long-term outcomes. On the other hand, patients undergoing consolidation-type TNT showed significant reduction in the odds of distant recurrence (27%), 90% increase in pCR at the cost of 86% higher likelihood of local recurrence [27]. Local recurrence rates though were available only for studies using SCRT in their consolidation regimen and this might also have influenced outcomes [13, 30]. Keeping in mind the importance of distant recurrence on patient prognosis [22], consolidation-type TNT regimens appear to be the preferable therapeutic option. Data supporting consolidation TNT are reported in two RCTs testing the optimal timing of TNT. Short-term results of CAO/ARO/AIO-12 and OPRA trials showed improved pCR and cCR, improved compliance to CRT and grade 3 to 4 toxicity rate reduction, when comparing consolidation and induction TNT [38, 39]. Recently, the CAO/ARO/AIO-12 trial long-term results confirmed consolidation TNT safety in terms of oncological endpoints, chronic toxicity, quality of life and stool incontinency [40]. However, long-term results in terms of 3-years disease free survival (DFS) from the phase 2 randomized OPRA trial are still awaited.

### Timing of surgery

The most advantageous timing of surgery following RT is not clear. Late surgery could be more technically demanding and results in worse TME quality and higher complications due to increased local fibrosis [41, 42]. Furthermore, non-responders could be at risk of both local and distant progression while waiting for surgery. On the contrary, longer free intervals increase effects of RT, augmenting chances to achieve pCR [43].

The TIMING trial, a phase II non-randomized trial, revealed that delivering consolidation TNT and delaying TME to up to 20 weeks after completion of TNT, increases pelvic fibrosis but does not increase the surgical technical difficulty nor the risk of surgical complications. It is not clear whether consolidation TNT or lengthening the chemoradiation-to-surgery interval led to pCR improvement [44].

In the most recent literature, time of surgery varied in the analyzed RCTs from 6 to 12 weeks, except for the RAPIDO trial in which surgery was performed 22 ± 2 weeks after the end of SCRT. The good results of the RAPIDO trial, in particular for what concerned the resection margins, suggest that longer intervals, whilst beneficial in terms of pCR, will not jeopardize surgical outcomes [30]. However, it is not clear whether pCR improvement is due to the administration of TNT or to the prolonged interval. Thus, these data will need validation before being fully applicable to patients at high risk of cancer progression.

### aCT

Several evidences suggest a lack of benefits of aCT in patients who have already received nCT [22–26]. Nevertheless, aCT was administered in 4 studies. Two of them provided aCT after surgery only in patients in the control arm [30, 37] but in the RAPIDO trial the choice of aCT based on CAPOX or FOLFOX and whether to use it or not was based on hospital and physician’s preference,. The other 2 studies administered aCT both in the control and experimental arms [31, 36]. Deng et al. administered 7 cycles of 5FU after surgery both in the control arm and in one of the experimental arms, while changing it to 6–8 cycles of mFOLFOX for patients who had not received neoadjuvant RT. Conroy et al. used adjuvant FOLFOX in both groups but patients in the experimental arm were delivered halved doses (6 vs 12 cycles) [36]. Given the high heterogeneity of protocols in use and the inter-variability amongst protocols, it is very difficult to draw sensible conclusions.

## Local disease control (LDC)

LDC can be pictured as a composite of different endpoints. More specifically, the main short-term endpoints for LDC, are pCR rates, nodal downstaging, percentage of R0 resection rate and percentage of lymphovascular and perineural invasion. The main long-term endpoint is local recurrence.

### pCR

Significance of pCR is well established due to its correlation with long-term oncological outcomes. Seven RCTs reported on pCR, including over 3000 patients. In particular Sauer et al. demonstrated improved 5-years DFS and OS in patients reaching pCR compared to incomplete pCR/non-responders (86% and 88% vs 63% and 76%, respectively) [45].

One meta-analysis, including 28 studies (of which 3 RCTs) confirmed a 39% increment in the odds of pCR (*p* = 0.01) [46]. The meta-analysis by Liu et al., which included 8 RCTs, showed an overall improved pCR. Sub-meta-analysis of available data underlined better results in terms of pCR when consolidation TNT is administered [32]. Of note, pCR improvement is particularly evident in the three most recent trials, perhaps due to progress in TNT regimens [30, 31, 36].

### Nodal down staging

Pathological N-stage after TNT (ypN) was collected by 6 out of 8 studies including 1866 patients, 931 in the experimental arm and 935 in the standard group. Patients achieving ypN0 in the experimental group were 685 vs 630 in the control (74% vs 67%). Overall, TNT does not seem to induce significant nodal down staging. However, sub-analysis of induction TNT had demonstrated that this strategy decreases likelihood of residual nodal disease [27].

### Resection limits

Resection limits were available for 2268 patients reported by 6 RCTs showing comparable R0 resections rates. R0 was achieved in 1102 out of 1225 patients (90%) in the experimental arm and 959 out of 1043 patients (92%) in the control arm. Two meta-analysis confirmed no statistically significant differences in TNT and standard arm for what concern the rate of negative resection margins [32, 46].

### Invasion (lymphatic, vascular and neural)

Lymphovascular and perineural invasion (PNI) are extremely important to tailor patient treatment due to the increased risk of both local recurrence and metastatic disease.

Unfortunately, they are reported only by the PRODIGE-23 study [36], which showed non-significant differences.

### Local recurrence rate

Local recurrence rate is the main long-term endpoint. TNT overall seems to offer improved local control; however local recurrence rates in the long term, are reported only by two RCTs. Results of the GCR-3 do not show any difference in the experimental arm compared to the standard treatment [37]. Unexpectedly, data from the RAPIDO trial shows an increased local recurrence rate for patients undergoing TNT despite the increase in pCR [30]. However, around 60% of patients had threatened/involved mesorectal fascia at diagnosis, but whether these patients accounted for most local failures remains uncertain [30].

Given the little data available on long-term DFS, it is impossible to conclude whether TNT could improve LDC.

As a word of caution, the meta-analysis by Kong et al. put an alert on consolidation regimens because they may threaten local control [27] however, this was not confirmed by the short-term results of the OPRA trial [39].

## Distant disease control

Distant recurrence rates still represent the leading cause of mortality for rectal cancer patients [18], thus affecting survival outcomes. The underlying molecular mechanisms are highly complex and seem to involve, for example, redox regulations [47–49], p53 family members [4, 50–57], nucleic acid regulators [58], hypoxia regulators [59] or Bcle family members [60–63]. Distant disease control includes different long-term endpoints: disease free survival (DFS), overall survival (OS) and distant recurrence rate.

### DFS and OS

Survival outcomes have been reported by 5 RCTs, without underling any statistically significant difference, despite the better local disease control [13, 30, 31, 36, 37]. On the contrary, meta-analyses comparing TNT to the standard of care, report homogeneous results regarding DFS improvement in patients undergoing TNT [27, 28, 32, 45, 46]. In particular, the meta-analysis of Riesco-Martinez et al. reported an 18% reduction in risk of recurrence (*p* = 0.01) and a 19% reduction in mortality at 3 years (*p* = 0.04) [46].

These results may well be the most important effect of TNT: in fact, traditional neoadjuvant multimodality treatment failed to improve patient survival [64]. Addition of oxaliplatin during standard CRT also had disappointing outcomes [65]. TNT holds the promise of providing tangible, long-term gains prolonging rectal cancer patient lives.

### Distant recurrence rate

Distant recurrence rate was reported only in 3 out of 8 RCTs. The GCR-3 and the POLISH II trial did not show any statistically significant difference between the experimental and standard arm [13, 37]. On the contrary, the RAPIDO trial showed a significant reduction in distant recurrence rate in favor of the TNT regimen (67% vs 81%) [30].

Data from 2 meta-analyses showed a reduction by 21,5%-27% in TNT treated patients [27, 46], confirming the RAPIDO trial results [30].

Nevertheless, data are still insufficient to establish whether TNT could improve systemic control disease.

## Toxicity/complications

### Chemo-related adverse effect

Whether TNT brings a significant rise in neoadjuvant treatment-related adverse effects (AEs) is controversial [66, 67]. Five studies reported AEs rate: four of them showed an increased number of AEs in patients treated with TNT [13, 34, 35, 37] but the PRODIGE-23 showed a statistically significant reduction of adverse events after the administration of neoadjuvant therapy [36].

The most common AEs included diarrhea, nausea, neutropenia and fatigue [28, 32]. All of these and also infectious complications, which were particularly relevant in the RAPIDO trial [30], are reported in the most recent meta-analyses demonstrating the higher risk of developing also serious (Grade III/IV) AEs for TNT patients [32, 45]. Moreover, the CAO/ARO/AIO-12 trial showed grade 3–4 AEs during CRT were more frequent in the induction group compared to the consolidation group [40].

### Surgical complications

As reported by almost all the RCTs, TNT does not appear to increase incidence of overall or severe post-operative complications (Clavien-Dindo) [32, 44, 45]. The Memorial Sloan Kettering Center study reported significant benefits such as earlier stoma closure (72% vs 9%) and a 25% higher rate of minimally invasive surgical procedures in TNT treated patients [28, 68]. Moreover, the TIMING trial collected surgeons’ experience, estimating technical difficulty of the operation, that does not seems to differ between different study groups, despite the increased pelvic fibrosis [44].

### Compliance

The difference in compliance to TNT and aCT, defined as the administration of at least 75% of the prescribed dose, favor the TNT regimen in most trials. Compliance to TNT ranges from 82 to 100% [44]. In the RAPIDO trial reported compliance to TNT was 84%, consistent with data present in literature, while compliance to CRT and aCT were 93% and 58% respectively. Reasons for not receiving aCT were to be found on difficulties due to surgery but also because of ypN0, pCR or patients’ refusal [30].

## Predictors of disease control

Among preoperative prognostic factors, the most important are clinical TNM stage (cTNM), enlarged lateral nodes, extramural vascular invasion, mesorectal fascia involvement and predicted circumferential resection margins (CRM) involvement. Data regarding cTNM reported by all RCTs did not show any difference between experimental and standard arms. Some authors noted how in the RAPIDO trial, tumor regression in terms of ypTNM was not as good with advanced (cT4 tumors) as with other cT stages, suggesting a possible weaker response to TNT by cT4 cancers [69, 70]. However, these considerations are purely speculative as there was no proper cTNM-based stratification.

Unfortunately, extensive data about other preoperative prognostic factors is missing. Five RCTs reported data regarding mesorectal involvement [30, 31, 33, 34, 37] and only one assessed vascular invasion [30]. Predictive CRM [35, 36] and lateral nodes involvement [30, 36] were described by two RCTs. None of these studies reported sub-analysis investigating whether these preoperative factors could be predictive of TNT response. Considering that all kinds of TNT importantly prolong time from diagnosis to radical surgery (which remains the mainstay of rectal cancer curative treatment) and may result in overtreatment for non-responders, it seems reasonable to focus upcoming studies on identifications of factors capable of predicting response to TNT and consequentially to tailor the best treatment for each patient.

## Conclusions

CRT seems to be the preferable option in case of high-risk local recurrence cancers. The choice of CT agents other than 5FU or capecitabine seems to be guided only by local policies. Consolidation TNT shows some advantages over induction regimen but differences in long-term survival are still required in order to clarify this highly debated issue. The best timing for surgery following RT is not clear; a minimum of 10 weeks seems an appropriate period to assess pCR and consolidation TNT may allow for extended durations between radiotherapy and surgery. However, timing of surgery apparently does not affect oncological outcomes. TNT increases AEs but does not appear to influence overall survival.

TNT seems to increase pCR and reduce distant recurrence rates. DFS and OS are homogeneously improved with TNT.

Future studies, aimed at evaluating the best regimen, should also investigate factors capable of identifying top responders to TNT in view of a tailored approach for precision oncology treatment.

## Data Availability

Not applicable.

## Abbreviations

aCT: Adjuvant Chemotherapy
AEs: Adverse Effects
CAPOX/XELOX: Capecitabine and Oxaliplatin
cCR: Complete Clinical Response
CRM: Circumferential Resection Margins
CRT: Chemoradiotherapy
CT: Chemotherapy
DFS: Disease Free Survival
FOLFIRINOX: 5FU/Folinic Acid, Irinotecan and Oxaliplatin
FOLFOX: 5FU/Folinic Acid and Oxaliplatin
LARC: Locally Advanced Rectal Cancer
LDC: Local Disease Control
nCT: Neoadjuvant Chemotherapy
npCR: Near-Complete Pathological Complete Response
OS: Overall Survival
pCR: Pathological Complete Response
PNI: Perineural Invasion
RCTs: Randomized Clinical Trials
RT: Radiotherapy
SCRT: Short-course Radiotherapy
TNT: Total Neoadjuvant Therapy
5FU: 5-Fluorouracil
